# High-performance prediction of epilepsy surgical outcomes based on the genetic neural networks and hybrid iEEG marker

**DOI:** 10.1038/s41598-024-56827-3

**Published:** 2024-03-14

**Authors:** Lipeng Sun, Chen Feng, En Zhang, Huan Chen, Weifeng Jin, Junming Zhu, Li Yu

**Affiliations:** 1 Second Clinical Medical School, Zhejiang Chinese Medical University, Hangzhou, China; 2 Department of Neurosurgery, School of Medicine, Second Affiliated Hospital, Zhejiang University, Hangzhou, China; 3 School of Medicine, Epilepsy Center, Second Affiliated Hospital, Zhejiang University, Hangzhou, China; 4 IDG/McGovern Institute for Brain Research, Beijing Normal University, Beijing, China; 5 State Key Laboratory of Cognitive Neuroscience and Learning, Faculty of Psychology, Beijing Normal University, Beijing, China; 6 Department of Physical and Environmental Sciences, University of Toronto, Toronto, Canada; 7 School of Pharmacy, Zhejiang Chinese Medical University, Hangzhou, China; 8 School of Basic Medical Sciences, Zhejiang Chinese Medical University, Hangzhou, China; 9 Qiushi Academy for Advanced Studies, Zhejiang University, Hangzhou, China; 10 Key Laboratory of Drug Safety Evaluation and Research of Zhejiang Province, Hangzhou Medical College, Hangzhou, China

**Keywords:** Genetic neural network, Surgical outcome prediction, Seizure onset zone, Hybrid marker, Model comparison, Epilepsy, Machine learning

## Abstract

Accurately identification of the seizure onset zone (SOZ) is pivotal for successful surgery in patients with medically refractory epilepsy. The purpose of this study is to improve the performance of model predicting the epilepsy surgery outcomes using genetic neural network (GNN) model based on a hybrid intracranial electroencephalography (iEEG) marker. We extracted 21 SOZ related markers based on iEEG data from 79 epilepsy patients. The least absolute shrinkage and selection operator (LASSO) regression was employed to integrated seven markers, selected after testing in pairs with all 21 biomarkers and 7 machine learning models, into a hybrid marker. Based on the hybrid marker, we devised a GNN model and compared its predictive performance for surgical outcomes with six other mainstream machine-learning models. Compared to the mainstream models, underpinning the GNN with the hybrid iEEG marker resulted in a better prediction of surgical outcomes, showing a significant increase of the prediction accuracy from approximately 87% to 94.3% (P = 0.0412). This study suggests that the hybrid iEEG marker can improve the performance of model predicting the epilepsy surgical outcomes, and validates the effectiveness of the GNN in characterizing and analyzing complex relationships between clinical data variables.

## Introduction

Epilepsy, a debilitating neurological disorder, affects an estimated 60 million people worldwide and approximately 30% of those affected suffer from drug-resistant epilepsy (DRE)^1^. Successful surgical intervention provide better seizure control and overall quality of life compared to medication^2^. However, the success rate of the epilepsy surgery was only 70% for adult^3^. Central to the success of surgical interventions is the accurate identification of the seizure onset zone (SOZ). By accurately identifying the SOZ, physicians can surgically target and remove or disrupt the brain regions generating these abnormal activities, effectively reducing the frequency and severity of seizures. Moreover, localizing the SOZ helps prevent damage to healthy brain tissue, preserving the patient’s neurological function and cognitive abilities to the greatest extent possible. Therefore, for patients with drug-resistant epilepsy, precise localization of the SOZ is fundamental and a critical step in surgical treatment. This process necessitates neuroimaging assessments and localization techniques to ensure optimal therapeutic outcomes and patient safety, ultimately improving the quality of life for individuals living with epilepsy^2,4^. However, it is difficult to identify SOZ due to the high complexity of the brain network and the variability of epilepsy manifestations^4,5^.

A number of EEG markers have been employed to localize the SOZ. These markers are mainly established based on frequency band power analysis, functional connectivity analysis, and graph theory. For example, High-frequency oscillations (HFOs), those higher than 100 Hz, have emerged as excellent surgical marker due to its contribution to the precise localization of SOZ^6–9^. In addition, functional connectivity analysis has also gained popularity as a strategy for identifying the SOZ. This approach allows researchers to map out the SOZ by observing alterations in brain activity pre-seizure and post-seizure and analyzing the functional connectivity of electrical signals across various brain nodes^10^. Pearson’s correlation coefficient is often regarded as a measurement marker of intracranial functional connection^11^. Recently, the principle of neural fragility, which hypothesizes that epilepsy results from the disruption of the brain’s stable connectivity, is leveraged to pinpoint the SOZ^12^. Moreover, graph theory is utilized to gauge alterations in brain network connectivity^13–15^. Some graph theory constructs, including eigenvector centrality and betweenness centrality, have been used to detect the SOZ^16,17^. Graph theory have also been applied to high-frequency oscillation analysis, and exhibits high performance in identifying the SOZ^18^. It should be noted that, however, most of the above studies only focus on a single marker and the precision of individual markers for predicting and delineating the SOZ is often restricted, given their basis on specific assumptions and the variable recognition efficiency tied to patient-specific traits and distinct datasets. Faced with the high complexity and variability of epilepsy manifestations, it is necessary to identify the most suitable model tailored to the characteristics of iEEG data. Hence, the incorporation of diverse markers and development of an effective mathematical model is of paramount importance.

In this study, by combining the hybrid iEEG markers, we developed a predictive neural network model based on genetic algorithms, which is GNN^19^. We compared its predictive capacity for surgery outcomes with that of six other mainstream machine learning methods, including logistic regression (LR), decision tree (DT), backpropagation neural network (BPNN), support vector machine (SVM), naive bayes model (NBM), and k-nearest neighbor (KNN)^20–23^. Specifically, based on the analysis of the iEEG data from 79 epilepsy patients, we selected seven exceptional markers from 21 SOZ identification markers, including neural fragility and 6 spectral-based features and combined them into a hybrid SOZ related marker. Leveraging the GNN model based on genetic algorithms, we formed an internal quantitative relationship between the hybrid marker characteristics and surgical outcomes in epilepsy patients. As the result, this model predicted surgery outcomes with an accuracy of over 94%, showing substantial benefit to clinical surgeries with ordinarily low success rates of 30–70%^3^. A series of comparisons revealed superior performance of the GNN over traditional machine learning models when utilizing both hybrid and single markers, and improved performance when hybrid markers was employed various machine learning models relative to single markers.

## Results

### Twenty-one SOZ markers

The fragility of neural nodes can be quantified using high-resolution EEG technology to identify the SOZ^12^. Centrality, a graph theory concept, is also used in this study. It describes the importance of each node in the network based on the correlation of different nodes. Two different modes of centrality are used in this study: degree and eigenvector centralities^15,16^, which describe the importance of nodes in various ways and help distinguish the seizure area from other areas. The centrality is calculated based on the correlation coefficient and coherence of EEG data. The correlation coefficient is applied to time domain signals, whereas coherence measures the relevance between node signals in the frequency domain. EEG data are transformed from the time domain to the frequency domain by Fourier transformation and then subjected to the spectrum analysis. Based on the six spectral-based features of EEG (such as the beta band ranging from 13 to 30 Hz and high-gamma > 90 Hz), the average values are taken in a small-time window to obtain the brain energy changes. Please refer to the Method section for the formulae used for calculating the aforementioned markers.

**Figure 1:**
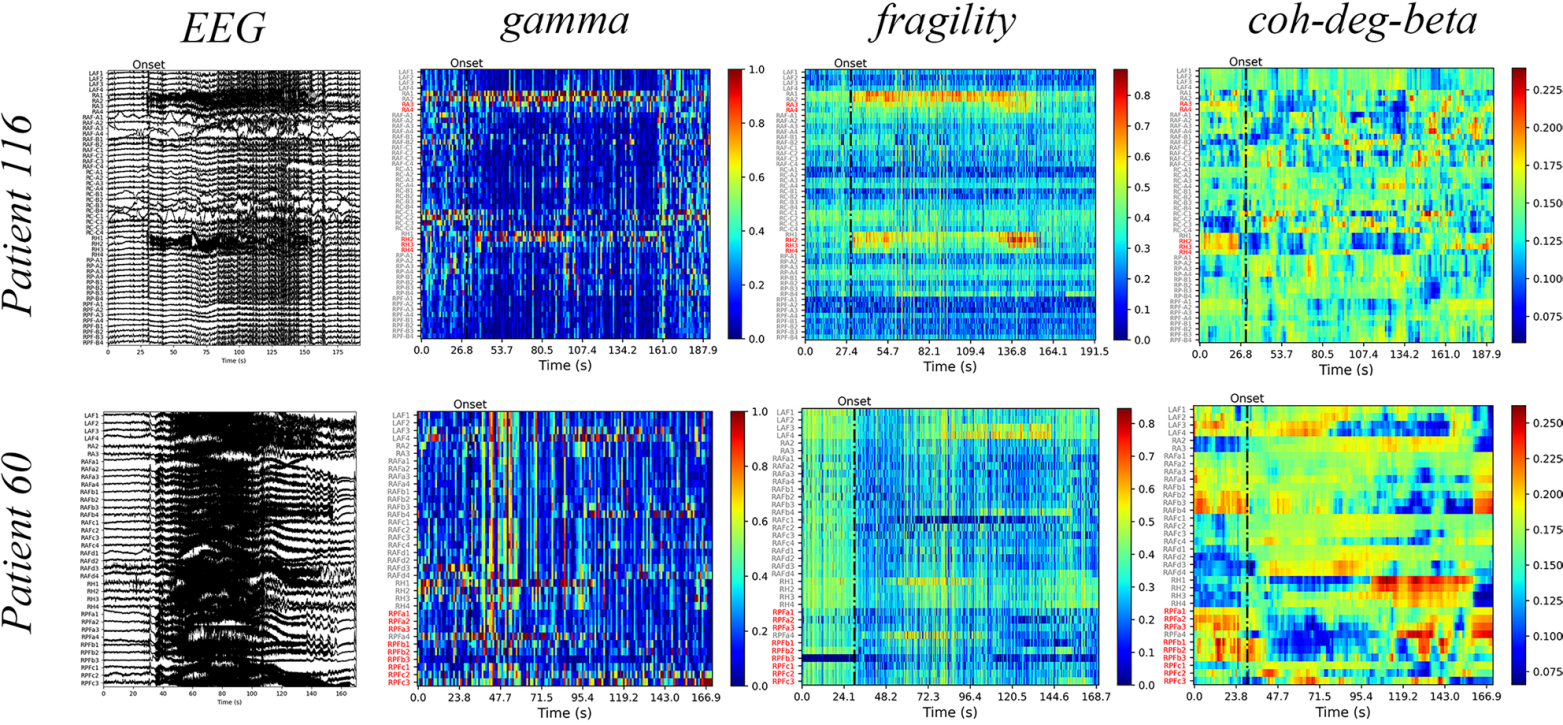
The performance of markers in the process of seizure. Patient 116 and patient 60 represent successful and failed surgical outcomes, respectively. The left shows the original EEG signal of a patient during an epileptic seizure, while the right displays the corresponding heat map, which reflects the performance of the gamma, fragility, and beta degree centrality markers. In the heat map, the vertical axis represents the electrode channels, with the red channels indicating those located within the surgical area, and the black dotted line indicating the time of seizure onset.

We illustrate the gamma, neural fragility, and beta band centrality of patients 116 and 60 (Fig. 1). The heat maps revealed that the surgical resection channel (red) for the successful patient 116 coincided with or is mostly located within the SOZ identified using the markers. Contrastingly, for patient 60 who had an unsuccessful surgery, the surgical area is not situated or is mostly not located within the SOZ identified using the markers. These findings highlight the importance of accurately identifying the SOZ for the surgery to be successful, as visually inspecting the original EEG alone may be insufficient to make such judgments. Additionally, the visualization through a heat map of markers, also known as EEG source imaging (ESI), assists clinicians in the selection of the surgical area^24^.

**Figure 2:**
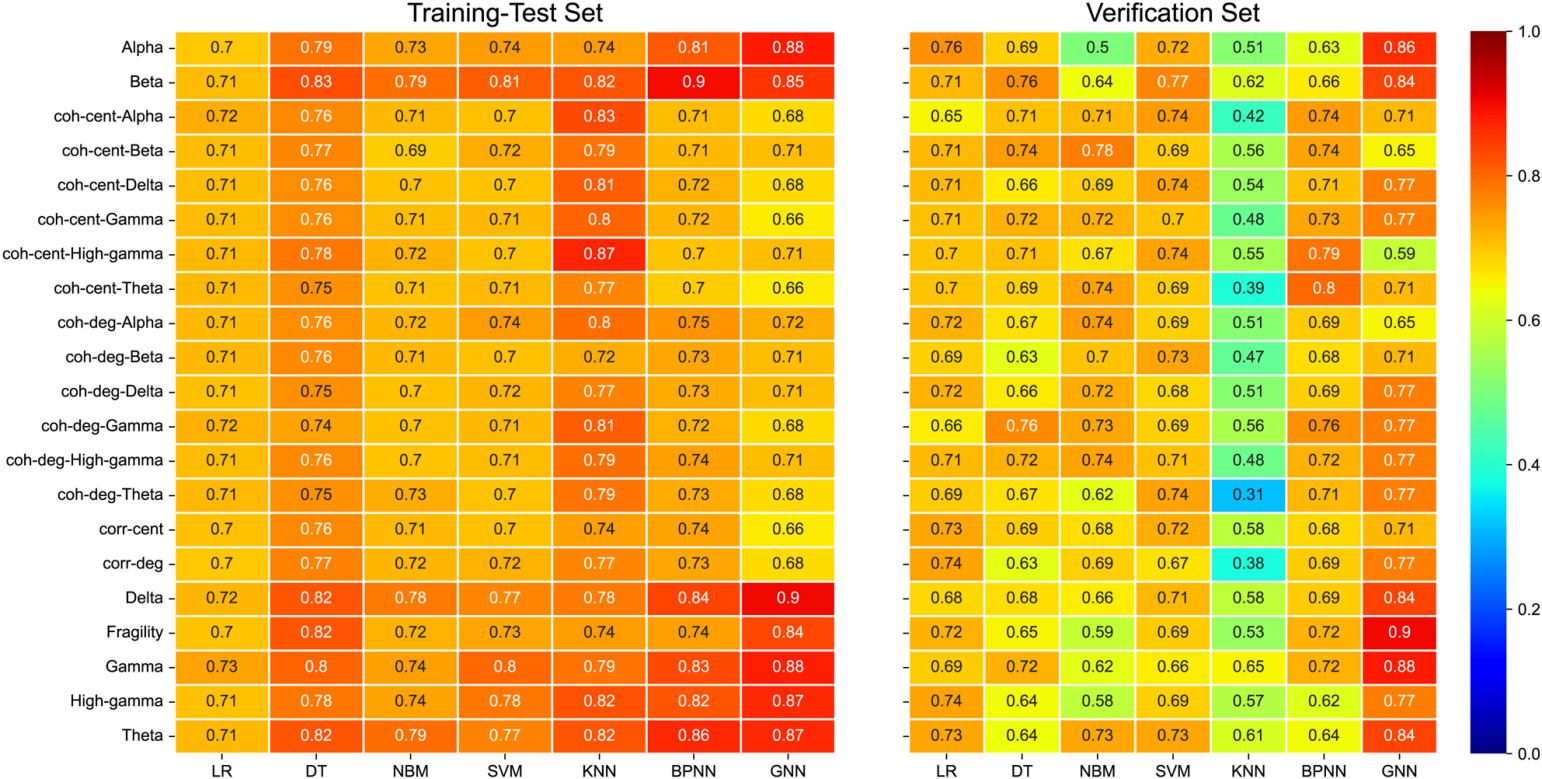
Average accuracy for training-test set (left) and verification set (right). Each combination is trained 10 times on a dataset of 79 patients, with the data split into 70% training and 10% test sets for each training and 20% data is fixed as verification set. The bottom labels represent the seven machine learning models, while the vertical labels represent the 21 markers. The number in each box indicates the average accuracy of 10 trainings, and its color corresponds to the rightmost color bar. The average accuracy of all combinations in the training set is 74.85%, while that in the test set is 68.13%. The highest accuracy rate for the entire dataset is obtained by the Gamma-GNN combination, which achieves an accuracy of 87.90%. Accuracy is the proportion of patients whose surgical results are correctly predicted by the model.

We combine the 21 markers with seven machine-learning models and separately trained them. To ensure optimal performance, we determine the range of the relevant hyperparameters through pretraining and set the lost weight in PyTorch to avoid sample imbalance^25^. Each combination in the dataset of 79 patients is trained 10 times and 20% data is fixed a verification set. During each training, 70% of the data is allocated to the training set and 10% is allocated to the test set. For each combination, the average accuracy rates on the training-test and verification sets are calculated (Fig. 2).

**Figure 3:**
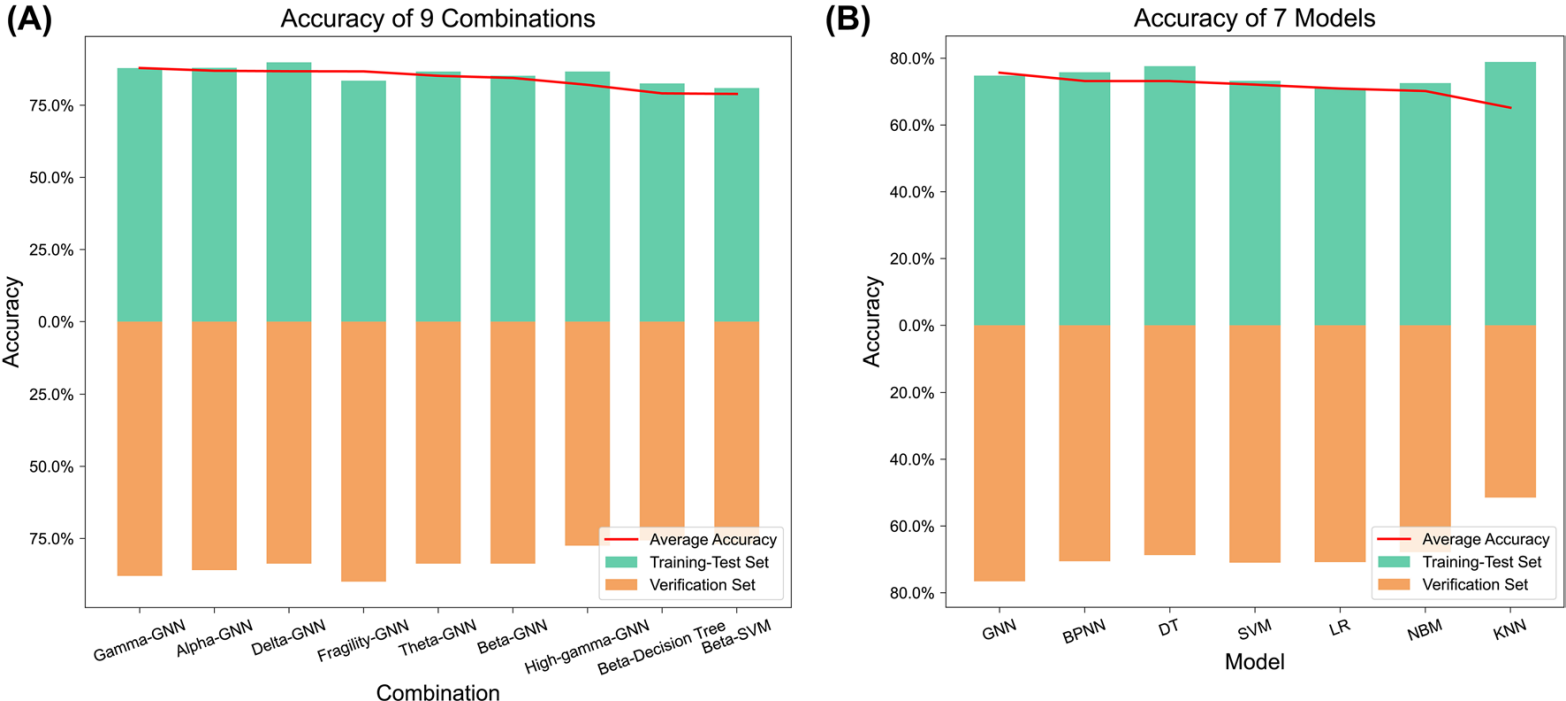
(**A**) Combinations with a top 25% accuracy on the training-test and the verification sets. After taking the intersection, we obtain 9 combinations and arrange their average accuracy rates in descending order. The red line in the figure represents the average accuracy of both sets. (**B**) The performance of the seven models in all combinations. The GNN model shows optimal performance, achieving average accuracy rates of 74.78%/76.58% on the training-test set/verification set. The traditional neural network is the sub-optimal model with accuracy rates of 75.81% and 70.55%.

According to the results obtained, we select combinations with a top 25% accuracy on the training-test and the verification sets. This result in 9 combinations intersecting (Fig. 3A). This method ensures that the chosen combinations have excellent performance on both sets, without exhibiting high accuracy in only one of them. Among these nine combinations, the accuracy of gamma-GNN with the best performance is 87.85/87.95% in the training test set/verification set, while that of the ninth beta-SVM model is 80.95%/76.87%. Meanwhile, 7 markers in these 9 combinations are commonly used markers, which include neural fragility and the averages of six frequency bands. Similarly, for each machine learning algorithm, these seven markers are also the best. Subsequently, these seven markers are used for further constructing hybrid markers. Additionally, the GNN performs well in all combinations, dominating the top seven. The average accuracy of the GNN is 74.78%/76.58% on the training-test set**/**verification set, while the sub-optimal model is 75.81%**/**70.55% (Fig. 3B).

### SOZ related hybrid iEEG marker

Directly combining the features of the seven selected markers result in a high-dimensional feature space that is not conducive to training subsequent machine learning models. This issue is addressed using LASSO regression, which selectively incorporates each feature into the model and reduces the dimensionality of the input features by controlling the coefficient to 0. This approach help avoid overfitting, thereby retaining six important variables (Supplementary Fig. 2).

### Performance of GNN combined with hybrid iEEG marker

**Figure 4:**
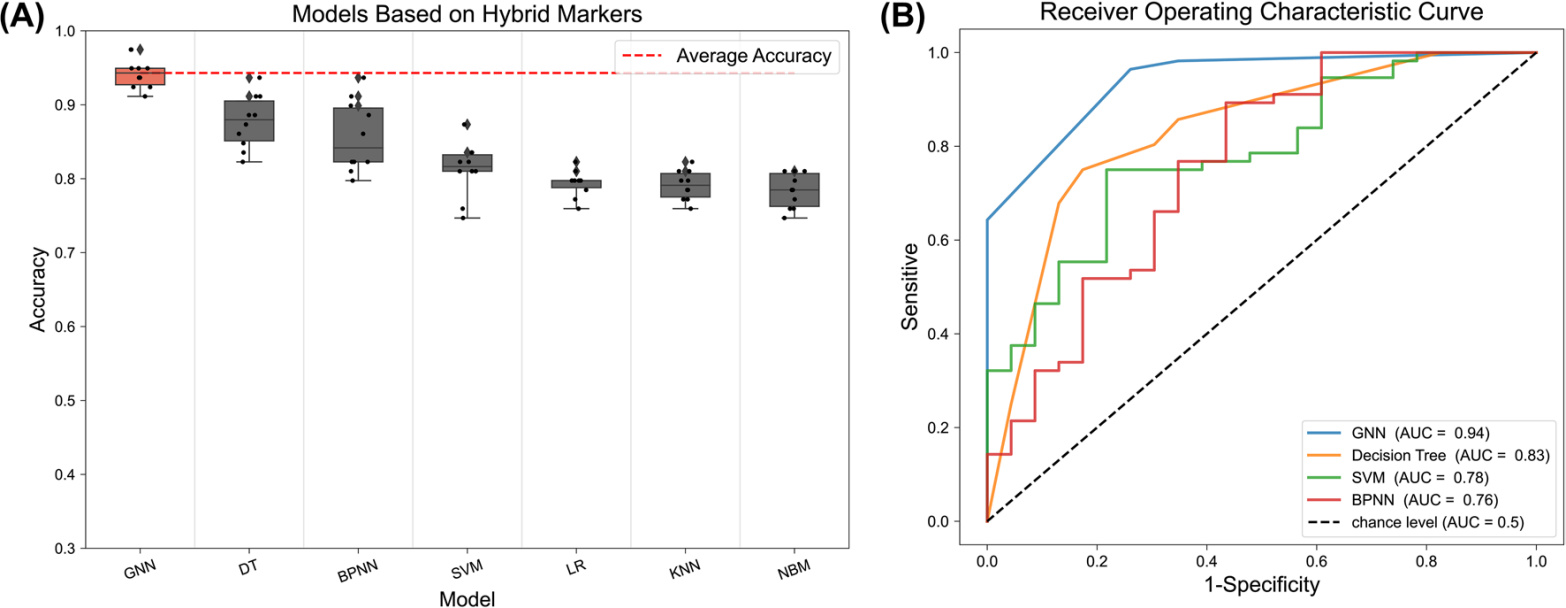
(**A**) Hybrid SOZ marker and seven models. The models are cross-validated 10 times, and the boxplots are sorted in descending order of their average accuracy. The red dashed line represents the average accuracy of the top-performing model, which is hybrid features and GNN with an average accuracy of 94.30%. The symbols on the boxplots represent the maximum value, upper quartile, median, lower quartile, and minimum value. The corresponding values for GNN are 97.47%, 94.94%, 94.30%, 92.72%, and 91.14%. The accuracy of GNN is significantly greater than decision tree (p = 0.0412 < 0.05, McNemar test). (**B**) ROC of GNN and the top 3 models with average accuracy. The horizontal axis represents the False Positive Rate (FPR, the ratio of patients who failed the surgery incorrectly predicted as success by a prediction model), and the vertical axis represents the True Positive Rate (TPR, the ratio of patients who succeeded the surgery that are correctly predicted as success by the model). The further from the pure chance line (diagonal), the better the model’s sensitivity and specificity. The black diagonal dashed line represents the random classification threshold. AUC stands for Area Under the ROC Curve and the closer the AUC is to 1, the better the model is at distinguishing between successful and failed patients. The ROC curve of GNN is significantly better than that of decision tree (p = 0.0461 < 0.05, Delong’s test).

We use the aforementioned hybrid SOZ markers as input for seven machine-learning models and use the surgical outcomes of patients as the output for investigating prediction efficacy. Each combination is trained 10 times, with training-test sets and verification sets. The average accuracy of GNN is 94.3% (Fig. 4A), which is significantly better than the decision tree of the suboptimal model by 6.59% (*P* = 0.0412; McNemar’s test; McNemar’s test can be used to compare the performance of two models on the same dataset)^26^. The maximum accuracy, upper quartile, median, lower quartile and minimum value in GNN training are 97.47%, 94.94%, 94.30%, 92.72% and 91.14%, while the highest accuracy of other models is only 93.67%. Receiver operating curves (ROC) are constructed to assess the classification ability of the models. The greater the area under the curve (AUC), the closer the curve is to the upper left corner (0,1). The GNN has the best performance with AUC = 0.94, which is significantly higher than that of the suboptimal model (13.25% higher; Fig. 4B; Delong’s test; maximum p-value of GNN and other models is 0.0461. The significance of the ROC curve is tested using Delong’s test^27^). Moreover, considering all single SOZ markers and machine learning models previously mentioned, the GNN based on hybrid marker still exhibit superior performance, which is 7.28% higher than the suboptimal combination (Gamma + GNN: 87.90%).

**Figure 5:**
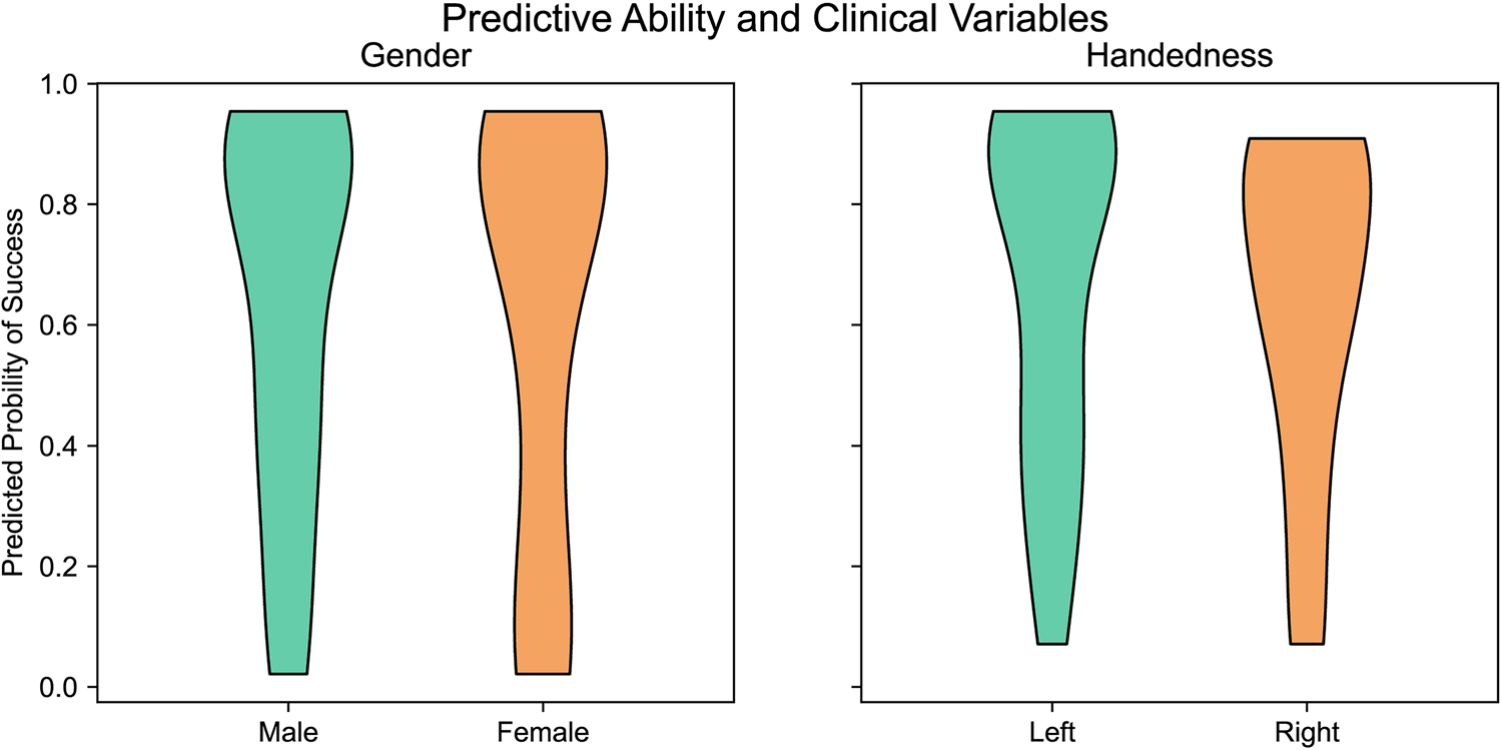
Relationship between predictive ability of hybrid + GNN and clinical variables. The y-axis represents the probability of a successful surgery prediction, indicating the likelihood of the model correctly predicting surgery success for a patient. There is no significant difference in the predictive ability of the model for patients with different sexes (p = 0.8245 > 0.05) and handedness (p = 0.3811 > 0.05, Mann–Whitney U test).

The predictive ability of the GNN based on hybrid markers remains unaffected by gender and the dominant hand (Fig. 5). The probability of predicting success represents the likelihood of a patient’s surgery is successful. A probability > 0.5 indicates that the model predicts the surgery to be successful and vice versa. The difference in the model’s predictive ability for patients of different sexes or handedness is insignificant (Mann–Whitney U test^28^; gender *P* = 0.8245 and handedness *P* = 0.3811).

## Discussion

In this study, we have undertaken an exhaustive comparison of common and SOZ related hybrid iEEG markers across a range of machine learning models, with a specific focus on their effectiveness in predicting successful epilepsy surgical outcomes. Our findings reveals that the GNN, underpinned by hybrid markers, achieves an impressive accuracy of over 94%, surpassing other models by more than 6.59% (*P* = 0.0412). This remarkable accuracy is achieved using the GNN model paired with hybrid markers, whether in singular or combined feature. In the single marker prediction, the spectral feature of high-gamma band has great performance, which is consistent with the current mainstream concepts such as the influence of high-frequency oscillation on epilepsy, suggesting that high-frequency band (> 90 Hz) cannot be ignored in the study of epilepsy origin^6–8^.

Epilepsy surgery is crucial for treating patients with drug-resistant epilepsy, and improving the success rate of surgery is of utmost importance. Even a small increase in prediction accuracy would represent a significant step forward. Numerous researchers have proposed various SOZ localization markers and epilepsy localization models to facilitate the assessment of the feasibility and efficacy of surgical plans by clinicians before surgery. Some of them also utilize machine learning models to predict surgical outcomes, achieving accuracy rates ranging from 60 to 95%^20,22,24^. Our accuracy surpasses the results reported in the majority of these research studies. Particularly noteworthy is that studies employing hybrid label-driven machine learning models consistently demonstrated strong performance on their own datasets, affirming the viability and effectiveness of this approach^23,29^. Our study presents a comprehensive “combination boxing” approach with an impressive prediction accuracy of 94.30%. With the incorporation of additional data, markers, or machine learning models, we can further enhance the precision of our predictions using this method.

Machine learning is an effective tool for recognizing data patterns with unclear mechanisms. However, training an unimproved machine learning model may yield inadequate results. Our GNN model differs from other mainstream machine learning models due to the utilization of genetic algorithm, which trains and optimizes neural network parameters through evolutionary processes, rather than relying solely on gradient-based optimization methods. This enables GNN model to effectively capture complex relationships within the data, enhancing its capability to handle nonlinear relationships and high-dimensional data. The significant improvement in prediction accuracy of GNN model is a result of the synergistic interaction between its unique neural network functionality and integrated genetic algorithm^19^. In this study, we compare the performance of a common neural network with that of a GNN. GNN outperformed in both single-marker and hybrid-marker scenarios. In particular, the GNN achieve an accuracy of 75.68% for a single marker and 94.30% for hybrid markers, whereas the common neural network achieved an accuracy of only 73.18% for a single marker and 85.70% for hybrid markers. Notably, the author of the fragility work also use deformation models of traditional random forests to predict surgical outcomes, whereas we propose a genetic algorithm-based neural network that can expand the range of machine learning models with efficient prediction ability^30^.

Furthermore, we discover certain counterfactual scenarios when we use this approach to review and analyze previous surgical resection plans.

**Figure 6:**
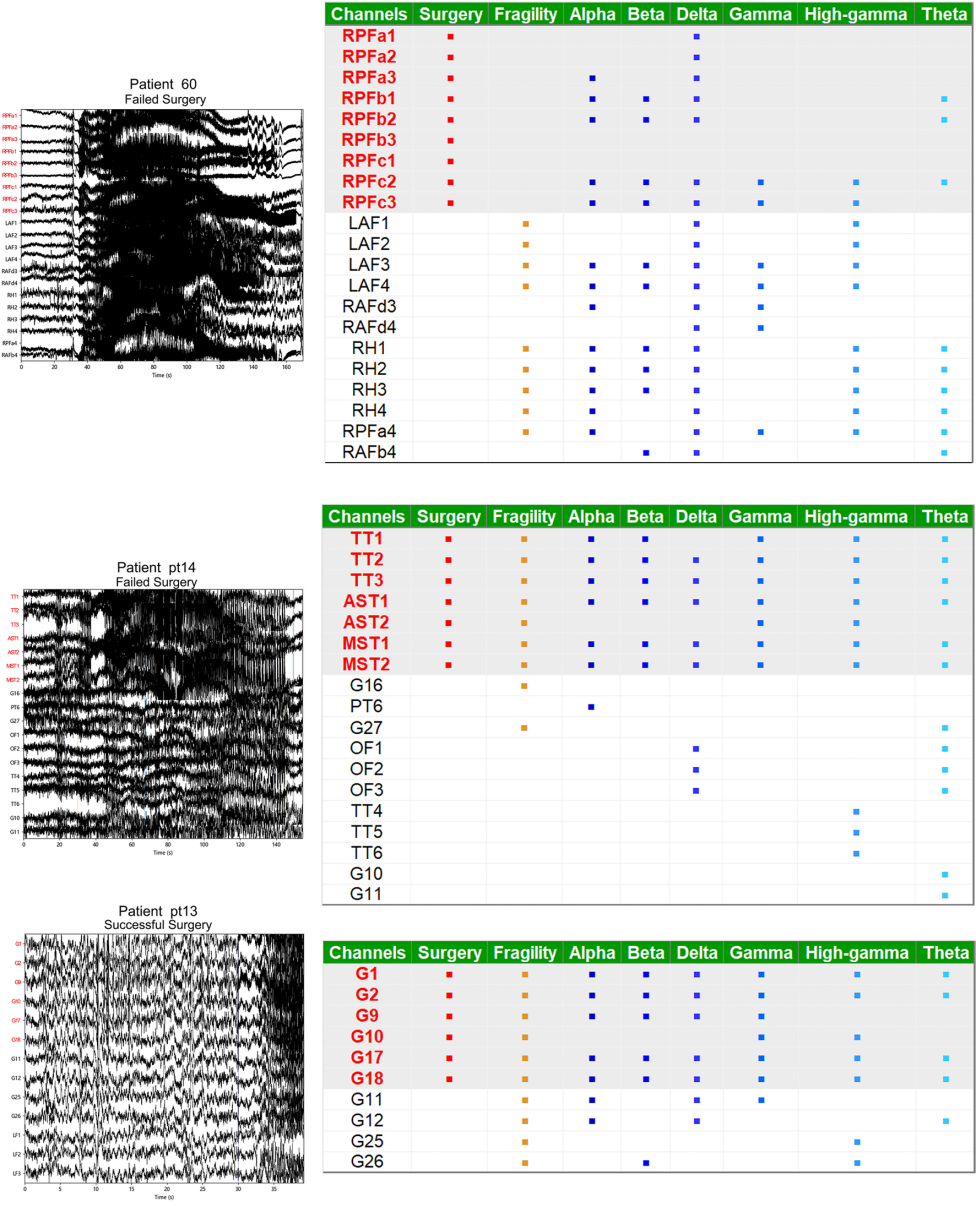
Retrospective analysis of patients’ surgery. Patient pt13 has a successful surgery and the model prediction is also successful. On the other hand, patient 60 has a failed surgery and the model prediction is also failed. Patient pt14 has a failed surgery but the model predicts success. The image on the left shows the original EEG from 30 s before the onset of the seizure. The table on the right displays the SOZ regional channels, including “**·**”, considered by seven markers. The column labeled “Surgery” indicates the channels that are included in the clinicians’ operating area, with the channels highlighted in red.

In Fig. 6, the left picture indicates the patient’s original EEG during the seizure, whereas the table on the right indicates the SOZ seizure areas identified by the seven markers. The surgical column shows the channels in the surgical area (indicated by the red font in the highlighted part of the table). Patient pt13 exhibit successful surgical outcomes, and our GNN model with hybrid markers predict the success of the surgery. The predictions of the seven markers closely matched the resection expectations of clinicians, indicating that the markers could simulate clinician judgment. Similarly, the surgery for patient 60 fails, and our model accurately predict surgical failure. We observed that the predictions of the markers are strikingly incongruous with the resection area of clinicians, suggesting that our model exhibited better judgment than the clinicians in some cases. For patient pt14, the predictions of markers matched the clinical viewpoints, and our model predicted successful surgery every time. Unfortunately, the surgery failed, suggesting that the failure is not attributed to inaccurate positioning. This failure may have been because of other factors such as postoperative rehabilitation and the formation of new lesions^2,5^. In summary, our GNN model with hybrid markers demonstrate SOZ localization abilities that are comparable to those of clinicians and provided accurate predictions of surgical results.

Currently, the inadequate availability of high-quality data and incomplete surgical information are major obstacles to accurately predicting epilepsy surgery outcomes. Our study data are from the open data repository^12,31^. These samples, originating from various studies, may be subject to factors such as collection methods, selection biases, or publication biases. While the sample size in our epilepsy SOZ recognition field may be deemed acceptable, it may still be insufficient for a large-scale model training purpose. The scarcity of data makes it challenging to apply large-scale models and use high-dimensional inputs, which in turn complicates the validation of the reliability of each SOZ marker in other study. Insufficient data not only relates to the current state of surgical treatment but also presents ethical considerations in medical practice. Furthermore, SOZ markers from different perspectives rely on their assumptions and unpublished data, complicating the replication of previous studies and making it difficult to evaluate the reliability of these markers.

Our manuscript delineates a study employing an approach combining multiple markers and machine learning models, applied to iEEG data to explore the potential for localizing SOZ and predicting surgical outcomes. Our findings stem from real clinical surgical samples across various studies, integrating 21 markers and 7 machine learning models. Considering factors such as sample representativeness, external validity, clinical relevance, and potential limitations affecting generalizability, we believe the credibility and persuasiveness of our research outcomes. However, in no way are we proposing that the direct clinical application of these algorithms or models. Instead, our discovery suggests that the integration of hybrid marker with the GNN model can more effectively identify SOZ and assist in predicting surgical outcomes, underscoring the need for caution when applying singular marker and machine learning models in clinical settings.

Our study has revealed a proliferation of various SOZ identification markers, offering new possibilities for clinical translation. We have proposed an innovative approach, termed the “combination boxing” method, which involves constructing hybrid markers and integrating refined machine learning models. This method holds promise in consolidating diverse markers to leverage their strengths, thereby enhancing identification accuracy and predictive performance. Moreover, we plan to investigate alternative data collection channels to maximize resource utilization, thus bolstering the credibility and impact of our study. Concurrently, we will engage in discussions regarding future research directions to further evaluate the applicability of our findings in clinical practice.

## Methods

### iEEG datasets and definition of surgical outcomes

We use the datasets collected by Lohith G and Adam Li groups (namely dataset 1 and 2 in this work), which is available from the open data website www.openneuro.org^12,31^. The detail information of the two datasets are summarized in Supplementary Table 1. The following data all indicate the electrodes contained in the surgery area, which are regarded as SOZ recognized by the clinicians, and other electrodes are regarded as SOZc. The SOZ related markers are calculated based on the period from 30 s before the seizure onset to the end for analysis. The selection of this period not only encompass the entire seizure event but also considered the iEEG information before the seizure. The definition of surgical success is the same in both datasets. After at least 12 months, patients who had surgical outcome of Engel I or ILAE 1–2 are marked as successful outcome and patients who had Engel II–IV or ILAE 3–6 are marked as failed outcome.

### Data processing

All the iEEG data are resampled to 512 Hz, notch filtered at 60 Hz with a cut-off window of 2 Hz, and band-pass-filtered between 0.5 Hz and the Nyquist frequency with a fourth-order Butterworth filter^12^. All markers from 30 s before the onset of seizure to the end are calculated by discrete time windows. And these time windows are set differently for different markers. Specifically, the time window of neutral fragility is 250 ms with 125 ms as the moving step, while that of other markers is 2.5 s with 500 ms as the step size^12,32^. Furthermore, in each time window, the calculation results of other markers except the related marks of feature vector centrality are normalized, to improve the accuracy of the machine learning models used subsequently.

### Calculation of 21 SOZ markers

Inspired by different epilepsy studies, the 21 markers in this study include the newly proposed neural fragility, frequency domain analysis in traditional research, and degree and eigenvector centralities in graph theory.

#### Neural fragility

Neural fragility is a positive indicator of how easily the brain is disturbed. The more easily the brain is disturbed, the higher the fragility. Recent research has demonstrated that neural fragility is also a marker of the SOZ. Specifically, by disturbing the stable brain network and calculating the fragility changes in each node during the seizure using iEEG data, the SOZ of the lesion can be located^12,32^. The connection model of the brain network adopts the idea of using linearity to fit nonlinearity, while a channel is considered as the sum of a portion of neurons, and its form is as follows:

1 $$\begin{array}{c}X\left(t+1\right)=\left(A+\Delta \right)X\left(t\right),\end{array}$$

where $X\left(t\right) \left(t\in {R}^{N}\right)$ represents the dynamic state of the brain and corresponds to the iEEG data with all channels in a time window (250 ms), matrix $A \left(A\in {R}^{N\times N}\right)$ represents the steady-state connection of the brain, and N is the number of iEEG channels. Seizure indicates that the brain has entered an unstable state and is disturbed, and the fragility is expressed by the model, which can be calculated using Eq. (2):

2 $$\begin{array}{c}\widehat{\Delta }=\left[{B}^{T}{\left(B{B}^{T}\right)}^{-1}b\right]{e}_{j}^{T},\end{array}$$

where, $B\left(\sigma ,\omega ,j\right)={\left[Im\left\{{e}_{j}{\left(A-\left(\sigma +j\omega \right)I\right)}^{-T}\right\},Re\left\{{e}_{j}{\left(A-\left(\sigma +j\omega \right)I\right)}^{-T}\right\}\right]}^{T}$, and normalization by $\Delta =\left({\text{max}}\widehat{\Delta }-\widehat{\Delta }\right)/{\text{max}}\widehat{\Delta }$^12,32^. Normalization is to better measure the differences in fragility of different parts of the brain at the same time.

#### 6 spectral-based features

The frequency bands for brain waves are strict, and different frequency bands correspond to different functional states of the brain. We divided the frequency bands into the following categorizations: Delta frequency band: 0.5–4 Hz; Theta frequency band: 4–8 Hz; Alpha frequency band: 8–13 Hz; Beta frequency band: 13–30 Hz; Gamma frequency band: 30–90 Hz; High-gamma frequency band: > 90 Hz. Notably, HFOs are included in the high-gamma frequency band.

We apply a one-dimensional Fourier transform to convert the original EEG data into frequency signals. The Fourier transform is a linear integral transform, which employs a sine wave to split the signal (Eq. 3):

3 $$\begin{array}{c}F\left(u\right)={\int }_{-\infty }^{\infty }f\left(x\right){e}^{-2\pi uxi}dx.\end{array}$$

Under the frequency signal, we consider the average value of each electrode as the spectral-based features in different time windows and then normalize them (Eq. 4). This normalization method is employed to prepare an electrode with a larger value that becomes a part of SOZ and an isotropic agent with the significance of other markers.

4 $$\begin{array}{c}F\left(u\right)=\frac{F\left(u\right)-{\text{min}}F\left(u\right)}{{\text{max}}F\left(u\right)-{\text{min}}F\left(u\right)}.\end{array}$$

#### Correlation coefficients and coherence

The correlation coefficients and coherence are calculated as the Eq. (5), respectively. And they are calculated separately within each frequency band. In the Eq. (5), (i, j) is the channel number, $Cov$ means calculating covariance, G means calculating cross spectral density^16,33^.

5 $$\begin{array}{l}\left\{\begin{array}{l}{Corr}_{ij}=\frac{Cov\left({x}_{i},{x}_{j}\right)}{{\sigma }_{{x}_{i}}{\sigma }_{{x}_{j}}}\\ {Coh}_{ij}\left(f\right)=\frac{{\left|{G}_{ij}\left(f\right)\right|}^{2}}{{G}_{ii}\left(f\right){G}_{jj}\left(f\right)}\end{array}\right..\end{array}$$

#### Degree centrality and eigenvector centrality

Centrality measures the importance of a node in a complex graph network. Both of degree centrality and eigenvector centrality have demonstrated good performance in epilepsy localization, and they are calculated as the Eq. (6), respectively^15,17^.

6 $$\begin{array}{l}\left\{\begin{array}{l}{DC}_{i}=\frac{\sum_{j=1}^{{N}_{i}}{x}_{ij}}{{N}_{i}-1}\\ {EVC}_{k}\left(i\right)=\frac{1}{\lambda \left(k\right)}\sum_{j}{\overline{Coh} }_{ij}\left(k\right)\cdot {EVC}_{k}\left(j\right)\end{array}.\right.\end{array}$$

### Feature extraction

Each marker first needs to undergo feature extraction to use it as an input for the model and is represented as a two-dimensional data matrix consisting of time and electrode channels. To facilitate feeding the features into machine learning models, we perform dimension reduction by the following two steps. First, to extract 20 quantiles, we regard the surgical resection area marked in the dataset as SOZ and the remaining as SOZc. We accumulate the marker values of the SOZ and SOZc channels in each time dimension and then extract 10 quantiles of each channel in the time sequence respectively to obtain 20 values (the first 10 are SOZ and the last 10 are SOZc)^12^. Secondly, for the principal component analysis. We obtain the principal components from 20 values using 85% as the threshold of cumulative contribution rate. In this way, we have achieved dimensionality reduction for each marker.

To construct hybrid SOZ markers, we combine the seven selected outstanding markers as the input of machine learning models. If these markers are directly merged, it will generate too many features, so we perform Lasso regression to select the important variables whose coefficients are non-zero as the model inputs. Lasso regression introduces an L_1_ regularization penalty term into the loss function as follows,

7 $$\begin{array}{c}Loss\left(\omega \right)=\sum_{i=1}^{N}{\left({y}_{i}-{\omega }^{T}{x}_{i}\right)}^{2}+\lambda {\Vert \omega \Vert }_{1} ,\end{array}$$

where $\omega$ represents the weight coefficients of each feature. Under this framework, it updates the weight coefficients of some features to zero in the linear regression process, thereby achieving the goal of feature selection.

### Genetic neural network

In this study, a genetic algorithm is applied to optimize the weight of the neural network instead of the back propagation (BP) algorithm of the traditional network. In the traditional BP algorithm training, the algorithm gradually adjusts weights along the direction of local improvement, resulting in the problem of local minima. This issue is avoided using a genetic algorithm based on biological evolution to initialize the neural network with several groups of different weights and to jump out of the local minima with a certain probability. This genetic algorithm is more likely to approach the global optimal solution^19^. Specifically, we apply this algorithm to train the weights between the layers of the single hidden layer neural network. We set the individuals of the population in the genetic algorithm as the weights of the neural network and defined the fitness function as $fitness={\text{max}}\left\{{MSE}_{train},{MSE}_{test}\right\}$, where ${MSE}_{train}$ and ${MSE}_{test}$ denote the mean square error on the training and test sets, respectively. Its main application process is explained as follows and the training process is depicted as a flowchart in Supplementary Fig. 1.

### Mainstream machine-learning models

The other six machine-learning models include logistic classification, decision tree, neural network, support vector machine, naive bayes model, and neighborhood algorithm. These six algorithms are all implemented by Python’s SK-learn^34^.

The training of each model is divided into the training set (70%), the test set (10%) and the verification set (20%, it can also be called lock-box set). In the training set, 20% cross-validation and weight adjustment of the loss function are employed to prevent over-fitting. After training, the accuracy of the model is calculated in the training-test and verification sets. All 147 combined models are trained 10 times, and the training-test sets are re-divided for each training session and the verification set is fixed. Finally, the average accuracy of the model in the training-test set and the average accuracy in the verification set are calculated. The training mode of the hybrid feature model is the same as above.

### Statistics analysis

McNemar’s test is employed to compare the surgical prediction accuracy among these models. This test is particularly suitable for comparing the performance of models using the same dataset^26^. Delong’s test is used to assess the significance of the ROC curve. This test helps determine whether there are significant differences in the performance of the models based on the ROC curve analysis^27^. Furthermore, the Mann–Whitney U test investigates whether there is any influence of gender and dominant hand on the prediction ability of the model. This test allows us to determine if there are any statistically significant differences in the model’s performance based on these factors^28^. A significance level of P < 0.05 is considered to indicate statistical significance. All the statistical analyses are conducted using Python version 3.9.

### Supplementary Information

Supplementary Information.

## Data Availability

We obtained data from publicly available databases openneuro.org. The datasets are provided Lohith G and Adam Li groups respectively (ds004100 (https://openneuro.org/datasets/ds004100) and ds003029 (https://openneuro.org/datasets/ds003029)). The use of the database adhered to the terms of service and ethical guidelines set forth by the database providers. The data were anonymized and handled in accordance with privacy protection protocols. We express our gratitude to the data contributors and database maintainers for making their data publicly accessible, enabling our research.
