# Supplementary material for: High-performance prediction of epilepsy surgical outcomes based on the genetic neural networks and hybrid iEEG marker
Source: Sci Rep. 2024 Mar 14;14:6198. doi: 10.1038/s41598-024-56827-3 (PMC10940588; doi:10.1038/s41598-024-56827-3)
Supplement: Supplementary file 1 — Supplementary Information. [file 41598_2024_56827_MOESM1_ESM.pdf]

Supplementary

Supplementary table 1: Summary of Datasets

Dataset	Patients	Average age	Gender (M/F)	Outcome (S/F)	Average channels (S/F)	Channels in surgical area (S/F)	Implant type
1	54	35.37	17/23	39/15	105.10/114.33	13.11%/9.38%	ECOG, SEEG
2	25	36.29	12/13	17/8	65.06/84.13	24.35%/19.07%	ECOG

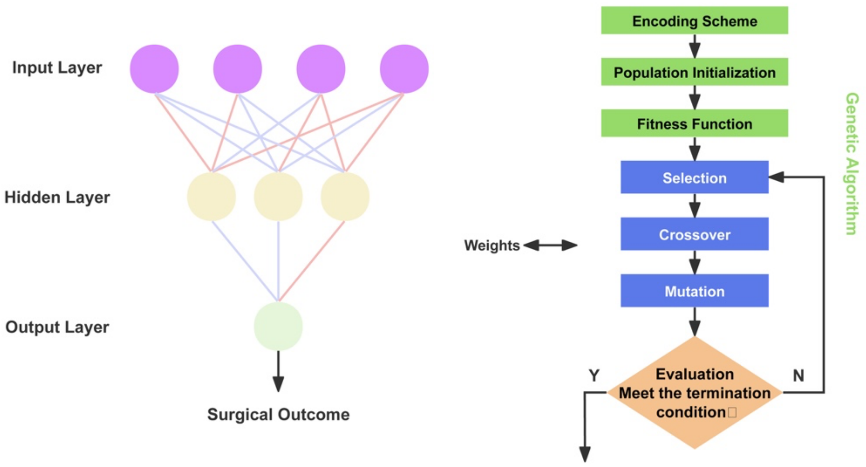

Supplementary figure 1. Framework of genetic neural network (GNN). Single hidden layer neural network, using genetic algorithm to optimize the weight. The input layer is the feature of the SOZ marker, and the output layer is the surgical outcome, with only two results: success or failure. By generating initial population, fitness function, coding individuals, and then continuously selecting new individuals, genetic algorithm jumps out of local optimization and gets the optimal weights.

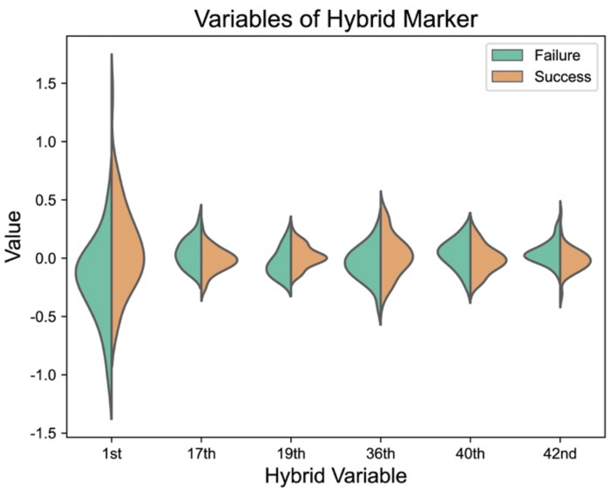

Supplementary figure 2. Six variables after Lasso regression. Lasso regression is performed after merging seven markers, and six variables (1st, 17th, 19th, 36th, 40th and 42nd) with coefficients not equal to 0 are retained as the feature of hybrid SOZ markers.

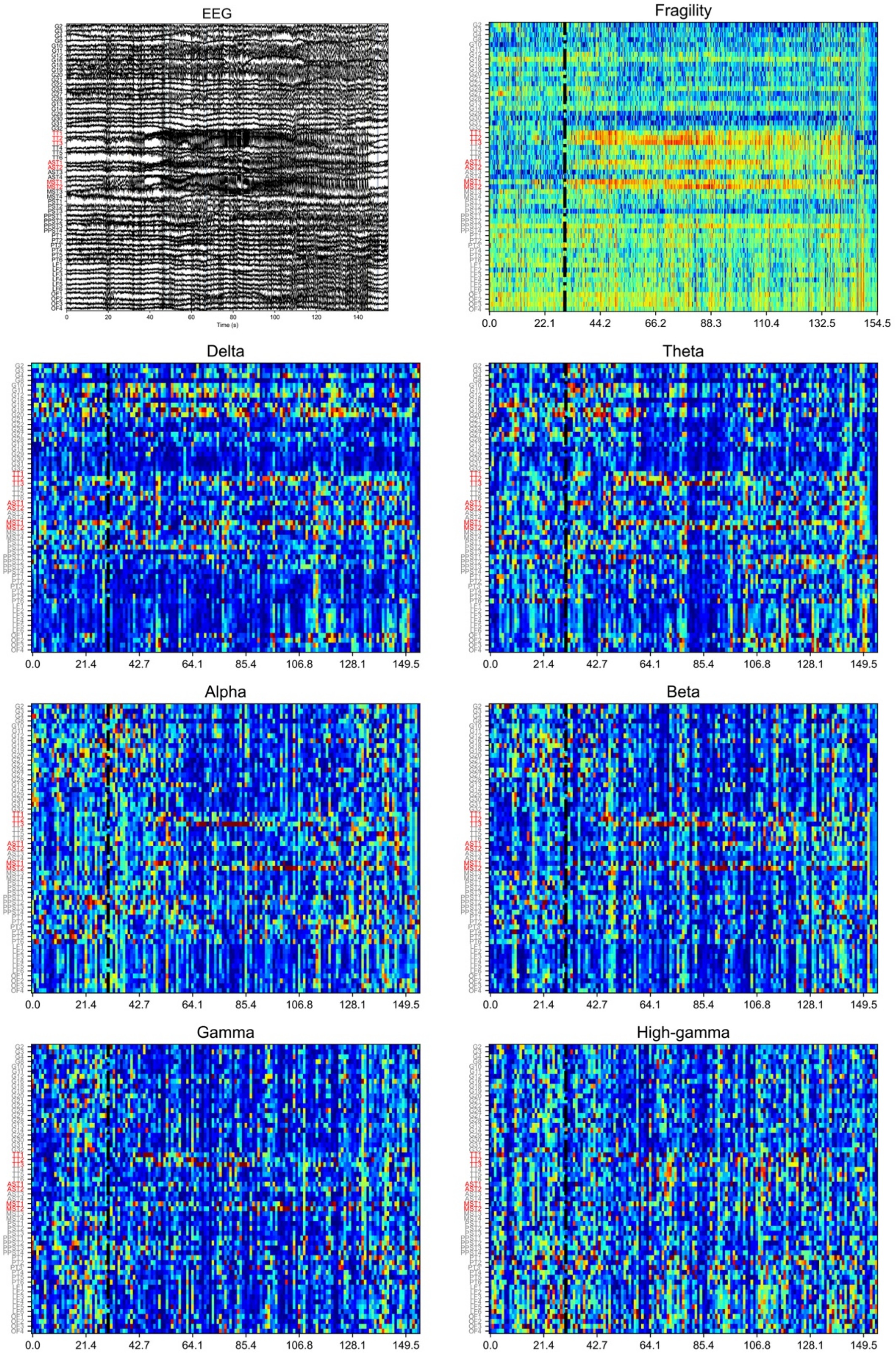

Supplementary figure 3. Seven marker heat map and the original EEG of patient pt14. The patient's actual surgery failed, but our model prediction is successful. The tips of the seven indicators are almost consistent with the views of clinicians, which indicates that the cause of the patient's surgical failure is not SOZ identification.
